# Heat to hypoxia cross‐adaptation: Effects of 6‐week post‐exercise hot‐water immersion on exercise performance in acute hypoxia

**DOI:** 10.1113/EP092726

**Published:** 2025-05-11

**Authors:** Patrick Rodrigues, Lydia L. Simpson, Justin S. Lawley, Heru S. Lesmana, Anne Hecksteden

**Affiliations:** ^1^ Institute of Physiology Medical University of Innsbruck Innsbruck Austria; ^2^ Institute of Sport Science University of Innsbruck Innsbruck Austria; ^3^ School of Sport and Human Movement University of Waikato Hamilton New Zealand; ^4^ Department of Physiology, Pharmacology and Neuroscience University of Bristol Bristol UK; ^5^ Department of Sport Coaching Universitas Negeri Padang Padang Indonesia

**Keywords:** altitude exercise performance, cross‐adaptation, hot‐water immersion, passive heating

## Abstract

Cross‐adaptation occurs when exposure to one environmental stressor (e.g., heat) induces protective responses to another (e.g., hypoxia). Although post‐exercise hot‐water immersion (HWI) induces heat acclimation, its potential to elicit cross‐adaptation remains unclear. This study evaluated the effectiveness of a 6‐week post‐exercise HWI intervention on exercise performance in hypoxia (O_2 _= 13%). Twenty healthy volunteers (28 ± 5 years; ${\dot{V}}_{O_{2}\mathrm{peak}}$ 47.4 ± 8.9 mL kg^−1^ min^−1^; 12 males, 8 females) completed interval cycling (4×4 min at 90 ± 5% maximal heart rate, 3×/week) followed by water immersion at either 34.5°C (control) or 42°C (HWI) for 40–50 min, five times per week. Following the 6‐week intervention, the post‐exercise HWI group exhibited lower resting heart rate (*P *< 0.01, *q* = 0.02; *d *= −1.32) and core temperature (*P *< 0.01, *q* = 0.001; *d *= −1.88) and elevated haemoglobin concentration (*P *< 0.01, *q* = 0.02; *d *= 1.38). Compared to the control group, the HWI group also showed greater improvements in time‐to‐exhaustion (TTE) trial (*P* and *q* < 0.01; *d *= 1.2) under hypoxia, but not in aerobic peak power (*P* = 0.03, *q* = 0.08; *d *= 0.86) or peak oxygen consumption (${\dot{V}}_{O_{2}\mathrm{peak}}$) (*P* = 0.04, *q* = 0.10; *d *= 0.82). Throughout the TTE, lower core temperature and tidal volume, with increased oxygen saturation and ${\dot{V}}_{O_{2}}$ were observed (*P* and *q* < 0.05). During hypoxic steady‐state exercise at 60% of ${\dot{V}}_{O_{2}\mathrm{peak}}$, the HWI group exhibited lower core temperature and higher peripheral oxygen saturation in hypoxia. No between‐group differences were observed in mean ${\dot{V}}_{O_{2}}$, respiratory exchange ratio, heart rate or rate of perceived exertion, nor in ${\dot{V}}_{O_{2}\mathrm{peak}}$ and aerobic peak power under normoxia (*P* and *q* > 0.05). In conclusion, post‐exercise HWI enhances maximal exercise performance under acute hypoxia, likely due to increased haemoglobin concentration, lower core temperature and improved respiratory efficiency.

## Abstract (highlights)

- **What is the central question of this study?**
  Can post‐exercise hot‐water immersion (HWI) induce a cross‐adaptation effect enhancing exercise performance in acute hypoxia?
- **What is the main finding and its importance?**
  Six weeks of daily post‐exercise HWI at 42°C (chest level, 40–50 min) elicits a cross‐adaptation effect in healthy, active cyclists. Post‐exercise HWI intervention improves exercise performance in acute hypoxia (13% O₂, simulating ∼4300 m altitude), as demonstrated by increased time‐to‐exhaustion at 80% of ${\dot{V}}_{O_{2}\mathrm{peak}}$. This improvement may be attributed to increased haemoglobin concentration, lower core temperature and enhanced respiratory efficiency (i.e., lower tidal volume, reduced ${\dot{V}}_{E}$ and increased absolute ${\dot{V}}_{O_{2}}$ and oxygen saturation).

## 1 INTRODUCTION

Heat acclimation provides beneficial adaptations that improve exercise performance in both hot and thermoneutral environments (Buchheit et al., 2011; Lundby et al., 2023; Philp et al., 2022; Racinais et al., 2014). Traditionally, heat acclimation involves exercise in an environmental chamber at 50–65% of peak oxygen uptake (${\dot{V}}_{O_{2}\mathrm{peak}}$), with temperatures between 33 and 40°C and 24–40% relative humidity (Lee et al., 2016; McIntyre et al., 2022; Zurawlew et al., 2016). Benefits of this approach include reduced cardiovascular strain and improved thermoregulation (e.g., lower core temperature, heart rate, blood pressure and improved sweating function) (McIntyre et al., 2022; Taylor, 2014). However, this method is often impractical due to logistical and financial constraints, as environmental heat chambers are generally inaccessible to the broader population and many semi‐professional and professional athletes. Thus, alternative, more practical and accessible interventions have been explored.

Post‐exercise passive heating, such as hot baths, has emerged as a promising method with similar benefits to traditional exercise heat acclimation. Forty minutes of hot‐water immersion (HWI) at 40°C after exercise has been shown to confer thermoregulatory adaptations for subsequent exercise in hot conditions. These adaptations include reductions in resting and exercise core temperature, rate of perceived exertion (RPE), heart rate, thermal sensation and higher sweating rate after 3 (Greenfield et al., 2021), 6 (McIntyre et al., 2021; Zurawlew et al., 2019, 2016) and 12 days (McIntyre et al., 2022) of intervention. Notably, a study showed that 6 days of post‐exercise HWI promotes greater thermal adaptations than conventional exercise‐based heat acclimation (60 min at 65% of ${\dot{V}}_{O_{2}\mathrm{peak}}$, 33°C, 40% humidity), including lower resting and exercise core temperatures, earlier sweat onset and an increased sweating rate (McIntyre et al., 2021). Furthermore, post‐exercise passive heating improves exercise performance in both hot and thermoneutral conditions (Philp et al., 2022; Zurawlew et al., 2016). These findings indicate that the combined thermal stimulus (endogenous and exogenous) provided by post‐exercise HWI may be an effective means to enhance both thermal adaptation and exercise performance.

Interestingly, research has suggested a cross‐adaptation effect between heat and hypoxia, whereby adaptation to one stressor (e.g., heat) elicits protective responses to another (e.g., altitude) (Fregly, 2010), reducing physiological strain (e.g., core temperature, heart rate and/or oxygen saturation) (Ely et al., 2014). Cross‐adaptation can occur at cellular, systemic, perceptual and performance levels. A recent systematic review found that most cross‐adaptation protocols involve 9 ± 3 heat sessions, generally inducing adaptations that reduce physiological strain during submaximal exercise in hypoxic conditions. These adaptations include lower RPE (Sotiridis et al., 2019; White et al., 2015), core temperature (Lee et al., 2016; Sotiridis et al., 2019; White et al., 2015), heart rate (HR) (Gibson et al., 2015; Lee et al., 2016; Sotiridis et al., 2019), ${\dot{V}}_{O_{2}}$ (Salgado et al., 2020; Sotiridis et al., 2019) and RER (Gibson et al., 2015), as well as increased peripheral oxygen saturation ($S_{pO_{2}}$) (Gibson et al., 2015; Heled et al., 2012; Lee et al., 2016) and delayed onset of blood lactate accumulation (Heled et al., 2012). However, maximal exercise performance in hypoxia after heat acclimation typically shows only small and trivial improvements (Willmott et al., 2024), with modest gains in peak power and time trials (Lee et al., 2016; Sotiridis et al., 2019; White et al., 2015) and no significant changes in ${\dot{V}}_{O_{2}\mathrm{peak}}$ (Heled et al., 2012; Sotiridis et al., 2019; White et al., 2015). The limited impact of these heat acclimation protocols on maximal exercise and athletic performance may be explained by their relatively short duration.

Although plasma volume expansion after short‐term heat acclimation is well‐documented, with increases of 4–15% (Périard et al., 2015; Willmott et al., 2024), studies have shown that this approach does not elevate haemoglobin concentration (6–10 days’ heat acclimation; Gibson et al., 2015; Lee et al., 2016; McIntyre et al., 2021; Zurawlew et al., 2019), which would benefit hypoxic exercise performance. Recent findings suggest that prolonged heat acclimation may be necessary to elicit such effects. Five weeks of exercise‐based heat acclimation (50–60‐min sessions, 5–6 times per week) increased haemoglobin mass and improved exercise performance, including enhanced ${\dot{V}}_{O_{2}\mathrm{peak}}$, peak power output and sustained power at 3 mmol L^−1^ [lactate] (Cubel et al., 2024; Lundby et al., 2023). Although these studies were conducted under normoxic and thermoneutral conditions, increased haemoglobin concentration could hold promise for performance in hypoxic conditions.

Accordingly, this study investigates whether a 6‐week heat acclimation intervention, implemented through post‐exercise HWI, enhances exercise performance under acute hypoxia more effectively than exercise training alone. Specifically, the study aimed to (i) assess exercise performance in a time‐to‐exhaustion trial under hypoxia following 6 weeks of post‐exercise HWI intervention; (ii) evaluate maximal aerobic performance (peak power and ${\dot{V}}_{O_{2}\mathrm{peak}}$) in normoxic and hypoxic conditions; and (iii) examine physiological responses during steady‐state exercise in both environmental conditions. In addition to a control group, the normoxic tests served as an environmental control. We hypothesised that (i) the 6 weeks of post‐exercise HWI would lead to greater improvements in exercise performance under hypoxia compared to exercise alone; (ii) participants from the HWI group would demonstrate lower physiological strain during steady‐state exercise; and (iii) performance gains and decreased physiological strain in hypoxic conditions would be more pronounced than in normoxic conditions.

## 2 METHODS

### 2.1 Ethical approval

This study was approved by the Ethics Board of the University of Innsbruck (No. 101/2023) and was conducted in accordance with the *Declaration of Helsinki* (2013). All participants received standardised information detailing the study's requirements and objectives to ensure awareness of the risk of their involvement before providing their written informed consent.

### 2.2 Participants

Twenty healthy and active university student volunteers (12 males and 8 females) completed this study. Initially, 22 participants were recruited and randomly assigned to either the post‐exercise HWI group or the post‐exercise thermoneutral‐water immersion (control) group, with sex matched between groups. During the intervention period, four participants dropped out: two females from the control group and two males from the HWI group. Reasons for dropout included severe flu symptoms, a knee injury from hiking, skin surgery and an ankle injury whilst skiing. To maintain a sample of 20 participants with balanced sex distribution, two additional participants were recruited: a female for the HWI group and a male for the control group (Table 1).

**TABLE 1:** Participant characteristics.

Variable	HWI group (*n* = 10)	Control group (*n* = 10)
Age (years)	28.3 ± 6.01	28.0 ± 3.88
Sex (male/female) (*n*)	6/4	6/4
Stature (cm)	177.6 ± 8.59	175.9 ± 8.34
Body mass (kg)	76.5 ± 15.1	75.7 ± 9.77
Absolute ${\dot{V}}_{O_{2}\mathrm{peak}}$ (L min^−1^)	3.57 ± 0.49	3.58 ± 0.80
Relative ${\dot{V}}_{O_{2}\mathrm{peak}}$ (mL kg^−1^ min^−1^)	47.7 ± 8.16	47.0 ± 9.76

*Note*: Data are presented as means ± SD.

Before beginning any testing, participants attended an orientation session where the study's design, objectives, testing protocols, exercise programme, potential risks and risk‐mitigation strategies were thoroughly explained. Participants also provided exercise and physical activity logs for the prior 2 months. Based on these logs, individualised exercise programmes were created, detailing how each participant would structure and complete their exercise routines in addition to the intervention in this study.

### 2.3 Study design

**FIGURE 1:**
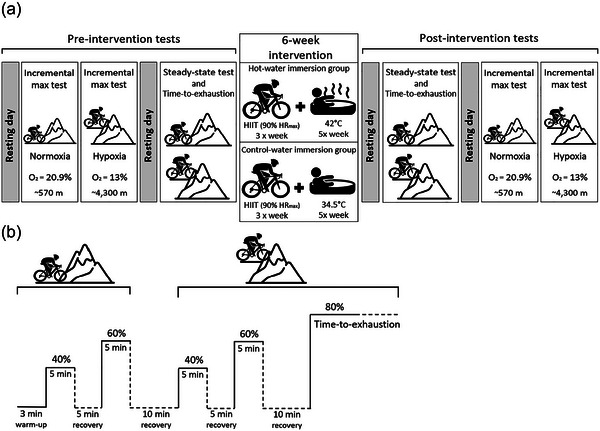
Study design. (a) Pre‐ and post‐intervention tests and the 6‐week intervention design. (b) Steady‐state exercise test and time‐to‐exhaustion trial protocols. Abbreviations: HIIT, high‐intensity interval training; HR_max_, maximum heart rate.

The pre‐intervention tests were conducted over three separate laboratory visits (Figure 1a). Initially, participants completed incremental maximal cycle ergometer tests under normoxic, then hypoxic conditions, across 2 days. After 24‐h rest, they performed two sets of steady‐state exercise tests at 40% and 60% of their peak aerobic power achieved during the incremental tests, first in normoxia and then in hypoxia. This was followed by a time‐to‐exhaustion trial in hypoxia at 80% of their maximum aerobic power (Figure 1b). Normoxic tests were conducted before hypoxic tests to prevent long‐lasting effects of acute hypoxic exposure on ventilation confounding factors, and the recovery periods (i.e., 5 and 10 min) were added to mitigate the effects of repeated tests.

The intervention period consisted of 6 weeks of cycling exercise training, with participants undergoing post‐exercise water immersion at either 34.5°C (control) or 42°C (HWI). After the 6‐week intervention period, participants first repeated the steady‐state tests and time‐to‐exhaustion trial, followed by the incremental maximal tests under normoxic and hypoxic conditions, each on separate days.

### 2.4 Exercise testing sessions

To control the effects of the circadian cycle on physiological responses, each participant performed the exercise test at the same time of the day (± 30 min). Female participants were scheduled for pre‐ and post‐intervention tests during the same phase of their menstrual cycle to control for potential hormonal effects on outcome variables. The pre‐ and post‐intervention tests were conducted approximately 8 weeks apart, and all female participants reported regular menstrual cycles ranging from 28 to 35 days. Participants were orientated to avoid alcohol consumption and not undertake strenuous exercise for 24 and 48 h prior to each exercise test. The post‐intervention test was conducted between 48 and 72 h after the last intervention session.

The incremental maximal tests were executed on a cycle ergometer (Cyclus2, Leipzig, Germany) to determine ${\dot{V}}_{O_{2}\mathrm{peak}}$ and peak aerobic power output. After a 3‐min warm‐up at 80 W, the incremental maximal test began at 80 W and increased by 5 W every 15 s. Participants were allowed to choose their preferred cadence between 90 and 120 rpm but were requested to maintain it at ±5 rpm once their preferred cadence was achieved, for the entire test. The incremental maximal test continued until volitional exhaustion was reached or the participant was no longer able to maintain the target cadence (<10 rpm for more than 5 s). ${\dot{V}}_{O_{2}\mathrm{peak}}$ was determined as the highest 30‐s average achieved during the test, and confirmed by reaching RER > 1.10, and RPE > 18 (6–20, Borg scale).

The steady‐state exercise test began in normoxia with a 3‐min warm‐up at 80 W, using the preferred cadence as per incremental test. Next, participants completed two sets of 5‐min fixed load cycling at 40% and 60% of their power output corresponding to ${\dot{V}}_{O_{2}\mathrm{peak}}$, with a 5‐min recovery period between sets (Figure 1b). After a 10‐min recovery, these sets of were repeated in hypoxia. Following a further 10‐min recovery, a time‐to‐exhaustion trial was conducted in hypoxia at 80% of their ${\dot{V}}_{O_{2}\mathrm{peak}}$. During each recovery period, participants were allowed to drink water ad libitum. The post‐intervention assessments for the steady‐state tests (40% and 60% of ${\dot{V}}_{O_{2}\mathrm{peak}}$) and the time‐to‐exhaustion trial (80% of ${\dot{V}}_{O_{2}\mathrm{peak}}$) were conducted using the same absolute workload levels adopted during the pre‐intervention assessments.

Throughout the steady‐state exercise tests and the time‐to‐exhaustion trials, various physiological parameters were continuously monitored, including ${\dot{V}}_{O_{2}}$, carbon dioxide production (${\dot{V}}_{CO_{2}}$), RER (${\dot{V}}_{O_{2}}$/${\dot{V}}_{CO_{2}}$), ventilation (${\dot{V}}_{E}$), $S_{pO_{2}}$, HR and core temperature.

### 2.5 Procedures and physiological measurements

Before the first exercise testing session, capillary blood samples were collected from the fingertip to assess haematocrit and haemoglobin concentrations (Swelab, Alfa, Stockholm, Sweden), and changes in plasma volume were estimated following the Dill and Costill (1974) method. For the exercise tests, the fraction of inspired oxygen ($F_{iO_{2}}$) was set at 0.21 for the normoxic conditions and reduced to 0.13 to simulate a hypoxic condition equivalent to an altitude of approximately 4300 m at a barometric pressure. Hypoxic air was obtained by an altitude generator (Hypoxico Everest Summit II hypoxic generator, New York, NY, USA).

${\dot{V}}_{O_{2}}$, ${\dot{V}}_{CO_{2}}$, RER and ${\dot{V}}_{E}$ were measured using a respiratory gas analyser (ML206, ADInstruments, Bella Vista, NSW, Australia) and a heated Pneumotach amplifier 1 series 1110 (Hans Rudolph Inc., Shawnee, KS, USA) connected to a gas mixing chamber system (MLA246, ADInstruments). Data analysis was performed using LabChart Software (Version 8, ADInstruments). Heart rate was recorded with a Polar belt monitor (Polar Electro, Kempele, Finland), and the RPE was assessed using the Borg scale (6–20 points). $S_{pO_{2}}$ was measured with a forehead pulse oximeter (Nellcor OxiMax M‐600x, Minneapolis, MN, USA), whilst core temperature was monitored using a disposable rectal probe inserted 12 cm beyond the sphincter (a‐line; Anandic, Switzerland).

Physiological transition thresholds were estimated using the first and second ventilatory thresholds (V1 and V2) during the incremental maximal tests under both normoxic and hypoxic conditions. To identify these thresholds, ${\dot{V}}_{O_{2}}$, ${\dot{V}}_{CO_{2}}$, RER and ${\dot{V}}_{E}$ data were averaged every 5 s throughout the test, transferred to an Excel spreadsheet and plotted. The ventilatory thresholds were identified by visualising the inflection points (or breakpoints) in the data plot. V1 was determined by the average breakpoint time across ${\dot{V}}_{E}$, RER and ${\dot{V}}_{E}/{\dot{V}}_{O_{2}}$, whilst V2 was identified by the second inflection across ${\dot{V}}_{E}$, ${\dot{V}}_{E}/{\dot{V}}_{O_{2}}$ and ${\dot{V}}_{E}$/${\dot{V}}_{CO_{2}}$ (Keir et al., 2022). Since each stage of the incremental test lasted 15 s, the identified breakpoint times for V1 and V2 were matched to the corresponding power output. Therefore, in this study, the ventilatory thresholds are presented at the corresponding power output.

During the steady‐state exercise tests, heart rate, ${\dot{V}}_{O_{2}}$, RER, ${\dot{V}}_{E}$, tidal volume and $S_{pO_{2}}$ data were analysed offline using LabChart Software. Once the physiological responses (i.e., heart rate, ${\dot{V}}_{O_{2}}$, RER, ${\dot{V}}_{E}$ and $S_{pO_{2}}$) had visually stabilised, reaching a plateau, a 2‐min window was selected, and values were averaged. RPE and core temperature were assessed during the last minute of each stage. For the time‐to‐exhaustion trials, physiological data were binned into 15‐s intervals at 25%, 50% and 75% time points of each trial, as well as the last 15 s representing 100% of time‐to‐exhaustion values.

Maximal exercise performance was assessed through peak power output and time‐to‐exhaustion. Peak power output was defined as the highest power (in watts) that the participant achieved and maintained for at least 80% of the stage duration (i.e., 12 s) during the incremental tests both in normoxia and in hypoxia. Time‐to‐exhaustion was measured in seconds and calculated as total power output generated throughout the trial. Although the exercise intensity (in watts) was kept constant between the pre‐ and post‐intervention tests, slight variations in cycling cadence could influence the cycling effort. Therefore, to determine the total power output, data recorded from the Cyclus2 ergometer during the test were downloaded. The cadence (RPM) was multiplied by the power (watts) every 0.5 s, then the entire column was summed and divided by 100.

### 2.6 Six‐week post‐exercise water immersion intervention

Participants in both groups engaged in a 6‐week intervention programme at the University Sports Science campus, involving three supervised cycling sessions and five water immersion sessions per week. The exercise sessions were performed on a stationary spin bike (Schwinn – 700IC, Vancouver, WA, USA), where participants completed high‐intensity interval training composed of four sets of 4‐min intervals at 90 ± 5% of their maximum heart rate (5‐s average), based on their incremental test in normoxia, with 4‐min intervals between sets. Heart rate was monitored using the Polar Beat app to ensure participants stayed within the targeted training zone.

Immediately following each exercise session, participants immersed themselves in water heated to either 42 ± 0.3°C (Rodrigues et al., 2020) or 34.5 ± 0.2°C up to the sternum level (xiphoid process), whilst arms remained outside. For both groups, the water immersion sessions were conducted in an inflatable bath five times a week. In the absence of an external heat pump, water temperature was manually regulated. A temperature probe with 0.1°C accuracy (the same used for core temperature assessment) was placed in the bath throughout the session. Hot water was added when the temperature dropped to 41.8 or 34.3°C, and cold water was added when it rose to 42.3 or 34.8°C. If the water level exceeded the xiphoid process, excess water was removed to ensure consistency in immersion depth. To progressively increase heat stress during the intervention, water immersion durations were set at 40 min for the first 2 weeks, 45 min for Weeks 3 and 4 and 50 min for Weeks 5 and 6. Participants were allowed to drink water ad libitum and had access to an electric fan to minimise dehydration and improve thermal comfort, respectively. Core temperature was measured in both groups during the first and final sessions of the 6‐week intervention at baseline, post‐exercise (pre‐immersion) and immediately after water immersion (i.e., 40‐min session).

On 2 days per week, water immersion sessions were undertaken without a corresponding high‐intensity interval training session. On these days, participants completed supervised water immersion sessions after their self‐directed training, which was conducted outside the University environment. Consequently, there was a gap of more than 1 h between their exercise and the bath sessions. As all participants were highly active, they were permitted to continue their regular physical activities (e.g., strength training, CrossFit, jogging, hiking, horse riding, handball, volleyball, football, skiing, snowboarding and yoga) on non‐exercise intervention days. They were instructed to maintain their usual activity level from the previous 2 months and to refrain from any new activities involving environmental stress (e.g., sauna, cold‐water immersion, altitude training). At the end of each week, participants submitted a report detailing all exercise, activities and volumes performed outside of the study to ensure adherence to the instructions and consistency in their training volume across the intervention period.

Each participant completed all 18 supervised exercise sessions, and participants from both groups completed between 26 and 30 water immersion sessions in total. Three water immersion sessions per week were the minimal requirement to remain in the study, and all participants complied with this criterion.

### 2.7 Statistical analysis

The primary analysis of this study compared score changes between groups (i.e., Δ vs. Δ) using an independent Student's *t*‐test. Additionally, within‐subject changes were assessed using a paired *t*‐test to determine the magnitude of changes within each group. The α level was set at 0.05. For physiological changes (within and between groups) during the time‐to‐exhaustion trials (at 25%, 50%, 75% and 100%), a two‐tailed *t*‐test was applied since the direction of the changes was unclear. When data normality was violated according to the Shapiro–Wilk test, the Wilcoxon rank test was used for paired samples. For independent samples, Welch's test was applied when normality was violated (Shapiro–Wilk test) but not homogeneity (Levene's test), and the Mann–Whitney *U*‐test was employed when homogeneity was not met. To account for multiple comparisons, we applied the Benjamini–Hochberg procedure in our primary analysis (Δ vs. Δ) to control the false discovery rate (FDR) at a threshold of 0.05. FDR‐adjusted *P*‐values (*q*‐values) were calculated, with results considered statistically significant if *q* < 0.05. For transparency, both the original *P*‐values and the corresponding *q* values are reported in the figures, tables and text. Additionally, effect sizes were calculated to provide a more comprehensive interpretation of the magnitude of changes, as recommended by the American Statistical Association (Wasserstein & Lazar, 2016).

The effect sizes are indicated by Cohen's *d* (± 95% confidence interval), with thresholds of 0.2, 0.5 and 0.8 for small, medium and large effect sizes, respectively. For non‐parametric tests (Wilcoxon or Mann–Whitney), the effect sizes are given as the rank biserial correlation (*r*_rb_), with ranges of 0.0–0.3, 0.3–0.5 and 0.5–1.0 for weak, moderate and strong relationships, respectively. For correlation tests between haemoglobin concentration and exercise performance (i.e., ${\dot{V}}_{O_{2}\mathrm{peak}}$, peak power output and time‐to‐exhaustion), Pearson's correlation test (*r*) was used or Spearman's test (rho) for non‐normal distribution. Correlation strength was categorised as follows: 0–0.19 indicates a very weak correlation, 0.2–0.39 weak, 0.4–0.59 moderate, 0.6–0.79 strong and >0.8 very strong. Statistical analysis was conducted using Jamovi software.

## 3 RESULTS

### 3.1 Resting values

**FIGURE 2:**
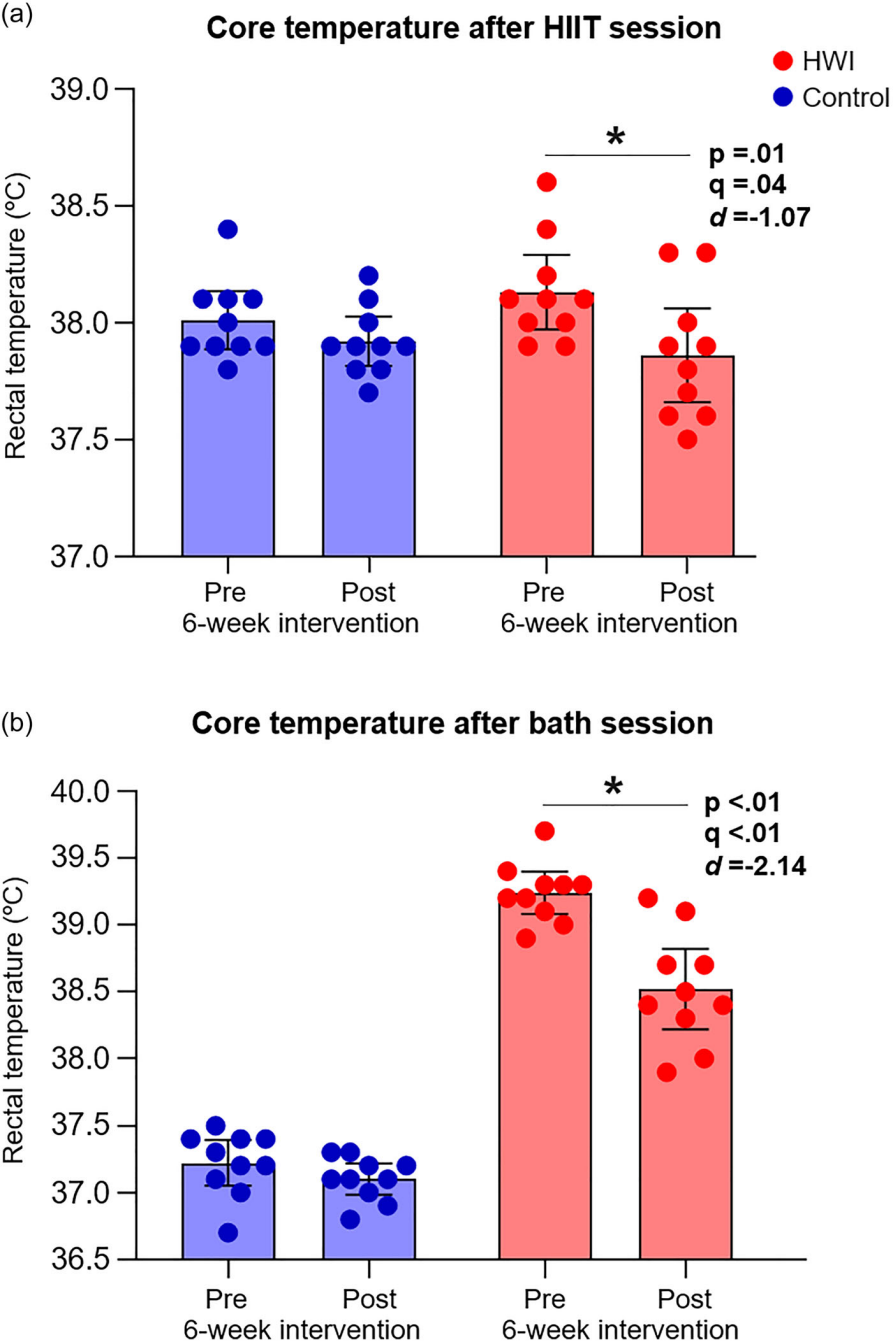
Changes in core temperature following the exercise training session (a) and the water immersion session (b) before (pre) and after (post) the 6‐week intervention. Data are presented as individual data and means ± 95% CI. HIIT, high‐intensity interval training; HWI, hot‐water immersion.

Following the 6‐week intervention, blood haemoglobin concentration increased in both groups, with a larger increase observed in the post‐exercise HWI group (5.33%) compared to the control condition group (2.03%) (Table 2). However, there was no significant difference in estimated plasma volume between groups, suggesting that the greater haemoglobin concentration was due to a greater increase in red cell mass. Body mass remained unchanged within and between groups. Both groups exhibited decreases in resting heart rate, but the decrease was significantly greater in the HWI group (HWI: −8.79%; Control: −3.87%). In addition, the HWI group presented a decrease in resting core temperature within and between groups (Table 2). The HWI group also demonstrated substantial decreases in core temperature immediately after the exercise training session (−0.27 ± 0.1°C, mean ± SD; *P* = 0.01, *q* = 0.04; *d* = −1.07 [−2.07, −0.27]) and after the water immersion session (−0.72 ± 0.1°C; *P* < 0.01, *q* < 0.01; *d* = −2.14 [−3.33, −1.44]) (Figure 2a,b). No significant changes in core temperature were observed in the control group after either the HIIT session (*P* = 0.04, *q* = 0.10) or the bath session (*P* = 0.07, *q* = 0.14) following the 6‐week intervention.

**TABLE 2:** Resting values of haemoglobin concentration, core temperature (rectal), heart rate and body mass before (pre) and after (post) the 6‐week intervention, alongside estimated changes in plasma volume following the intervention.

	Paired samples test			Delta vs. delta	
Parameter, group	Pre	Post	*P*‐value; Cohen's *d* [95% CI]	Mean difference	*P*‐value, *q*‐value Cohen's *d* [95% CI]
Haemoglobin (g dL^−1^)					
HWI	14.2 ± 1.40	15.0 ± 1.40	***P* < 0.01**; *d* = 0.58 [0.25, 0.97]	0.81 ± 0.45	***P* < 0.01, *q* = 0.02**; *d* = 1.38 [0.43, 2.34]
Control	14.5 ± 1.04	14.8 ± 0.99	***P* < 0.01**; *d* = 0.30 [0.13, 0.67]	0.30 ± 0.26	
Δ Plasma volume (%)					
HWI				2.09 ± 0.10	*P* = 0.47, *q* = 0.55; *d* = 0.03 [−1.06, 1.17]
Control				2.09 ± 0.06	
Rectal temperature (°C)					
HWI	37.3 ± 0.27	37.1 ± 0.29	***P* < 0.01**; *r*_rb_ = 0.38 [0.05, 0.65]	−0.22 ± 0.12	***P *< 0.01, *q* = 0.001**; *d* = −1.88 [−2.75, −0.92]
Control	37.2 ± 0.28	37.2 ± 0.21	*P* = 0.68; *d* = 0.08 [−0.28, 0.43]	0.02 ± 0.13	
Heart rate (beats min^−1^)					
HWI	73.9 ± 6.79	67.4 ± 5.52	***P* < 0.01**; *d* = −0.33 [−0.80, −0.06]	−6.50 ± 1.96	***P *< 0.01, *q *= 0.02**; *d* = −1.32 [−2.62, −0.15]
Control	74.9 ± 8.18	72.0 ± 9.10	***P* = 0.01**; *d* = −1.05 [−1.48, −0.70]	−2.90 ± 3.31	
Body mass (kg)					
HWI	76.5 ± 15.1	76.4 ± 15.3	*P*= 0.97; *d* = −0.00 [−0.07, 0.07]	−0.02 ± 1.76	*P* = 0.48, *q* = 0.56; *d* = 0.34 [−0.64, 1.26]
Control	75.7 ± 9.77	75.3 ± 10.3	*P* = 0.08; *d* = −0.04 [−0.12, 0.01]	−0.48 ± 0.77	

*Note*: Data are presented as means ± standard deviation (SD). Values in bold indicate a significant difference.

### 3.2 Exercise performance tests

**FIGURE 3:**
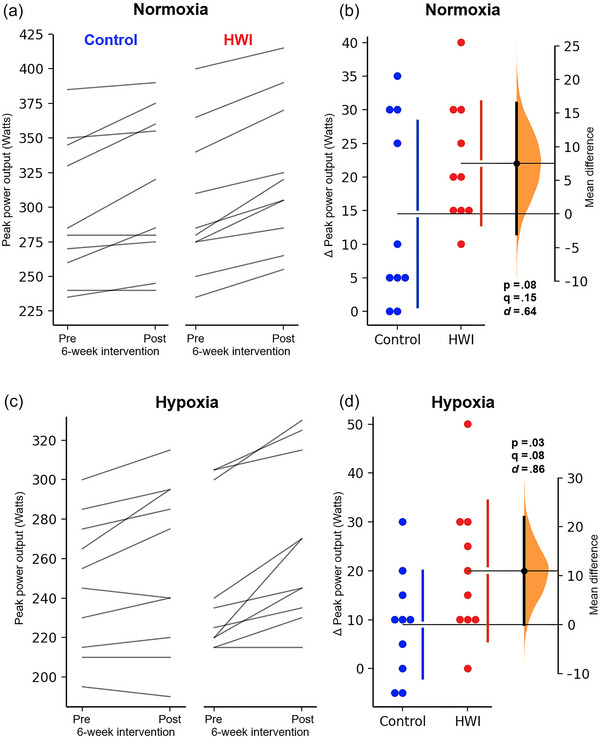
Changes in peak power output under normoxic (a, b) and hypoxic (c, d) conditions before (pre) and after (post) the 6‐week intervention. Data are presented as individual paired observations (within groups; a, c) and by a Gardner–Altman estimation plot for observations between groups (Δ vs. Δ; b, d). The changed score observations from both groups are plotted on the left and the mean difference is plotted on the right as a bootstrap distribution, mean ± 95% CI. The graphs were created using estimationstats.com (Ho et al., 2019). HWI, hot‐water immersion.

**FIGURE 4:**
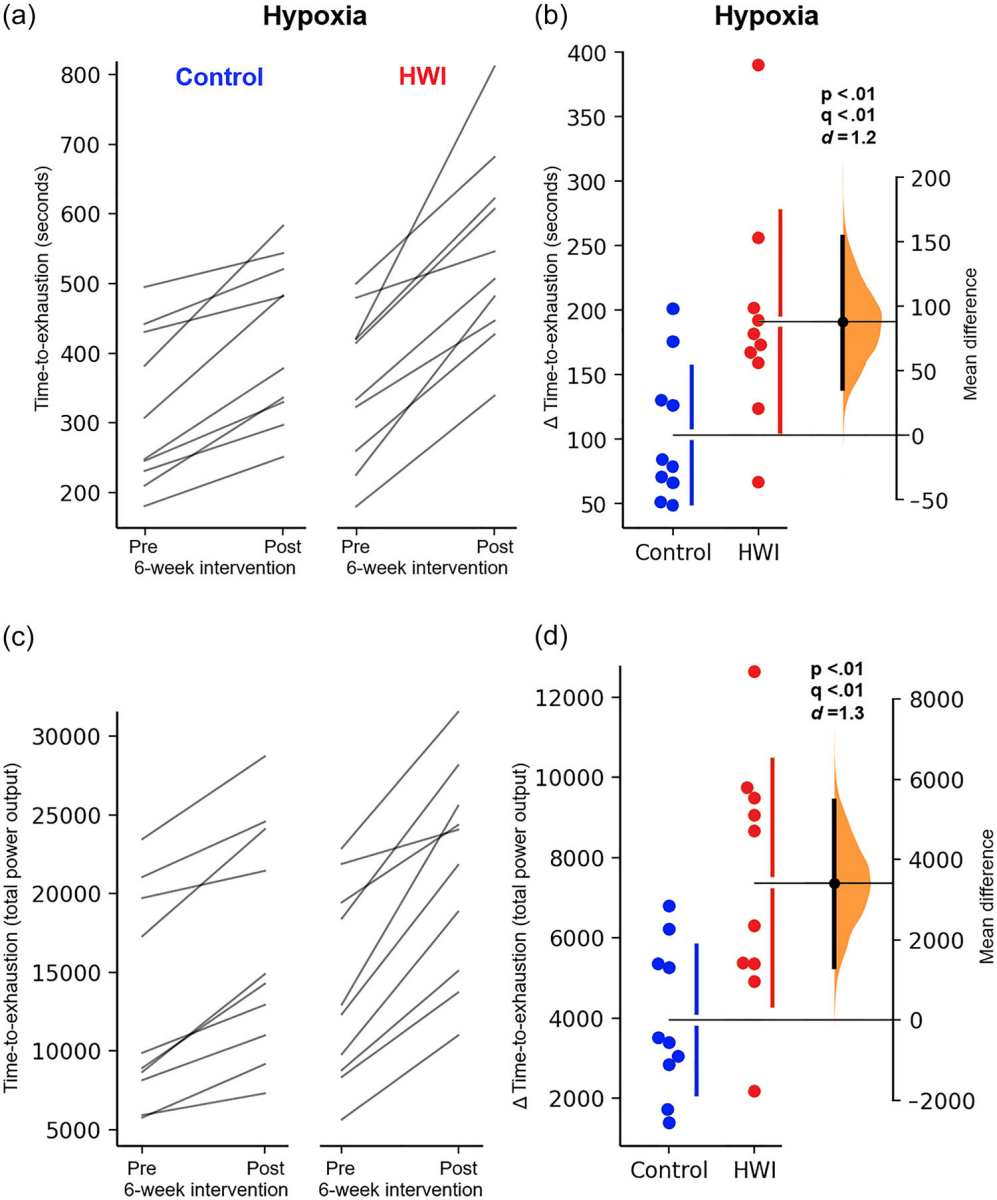
Changes in time‐to‐exhaustion trial at 80% of ${\dot{V}}_{O_{2}\mathrm{peak}}$ under hypoxia conditions in seconds (a, b) and in total power output (c, d) before (pre) and after (post) the 6‐week intervention. Data are presented as individual paired observations (within groups; a, c) and by a Gardner–Altman estimation plot for observations between groups (Δ vs. Δ; b, d). The changed score observations from both groups are plotted on the left, and the mean difference is plotted on the right as a bootstrap distribution, mean ± 95% CI. The graphs were created using estimationstats.com (Ho et al., 2019). HWI, hot‐water immersion.

Both groups improved the peak power output during the incremental maximal test under normoxic and hypoxic conditions (Normoxic, HWI: 6.79%, Control: 4.79%; Hypoxic, HWI: 7.46%, Control: 3.50%) (see Table 3 for means ± SD). However, no significant between‐groups differences were observed in normoxic (*P* = 0.08, *q* = 0.15; *d* = 0.64 [−0.31, 1.63]) or hypoxic conditions (*P* = 0.03, *q* = 0.08; *d* = 0.86 [−0.13, 1.75]) following the 6‐week intervention (see Figure 3 for paired individual data and changed scores [Δ vs. Δ]). Both groups also improve exercise performance during the time‐to‐exhaustion trial under hypoxia (Table 3), with the HWI group demonstrating a greater enhancement in both absolute duration (HWI: 34.9%, Control: 24.5%; *P* < 0.01, *q* < 0.01; *d* = 1.23 [0.15, 2.01]) and total power output (HWI: 34.4%, Control: 23.5%; *P* < 0.01, *q* < 0.01; *d* = 1.35 [0.28, 2.25]) (Figure 4).

**TABLE 3:** Maximal exercise performance test results before (pre) and after (post) the 6‐week intervention, including peak power output during the incremental maximal test and time‐to‐exhaustion (TTE) trial results in seconds and in total power output.

Parameter, condition, group	Pre	Post	*P*‐value; Cohen's *d* [95% CI]
Peak power output (W)			
Normoxia			
HWI	302 ± 52.2	324 ± 53.0	***P* < 0.01**; *d* = 0.42 [0.24, 0.67]
Control	298 ± 51.2	313 ± 54.8	***P* < 0.01**; *d* = 0.27 [0.10, 0.51]
Hypoxia			
HWI	248 ± 39.0	268 ± 41.8	***P* < 0.01**; *d* = 0.49 [0.28, 0.95]
Control	248 ± 34.7	257 ± 42.2	***P* = 0.01**; *d* = 0.23 [0.06, 0.49]
TTE at 80% of ${\dot{V}}_{O_{2}\mathrm{peak}}$ (seconds), hypoxia			
HWI	356 ± 109	547 ± 138	***P* < 0.01**; *d* = 1.54 [1.02, 1.92]
Control	317 ± 111	420 ± 116	***P* < 0.01**; *d* = 0.91 [0.58, 1.37]
TTE at 80% of ${\dot{V}}_{O_{2}\mathrm{peak}}$ (total power output), hypoxia			
HWI	14055 ± 6156	21422 ± 6634	***P* < 0.01**; *d* = 1.15 [0.74, 1.74]
Control	12891 ± 6744	16841 ± 7339	***P* < 0.01**; *d* = 0.56 [0.36, 0.83]

*Note*: Data are presented as means ± SD. Values in bold indicate a significant difference.

Significant improvements in absolute and relative ${\dot{V}}_{O_{2}\mathrm{peak}}$ were observed for both groups in normoxic and hypoxic conditions after the 6‐week intervention. However, no significant between‐groups differences were observed (Table 4). Similarly, for ventilatory thresholds, both groups experienced improvements (i.e., delays) in V1 under hypoxia and V2 under both normoxia and hypoxia, yet no between‐groups differences were detected (Table 4).

**TABLE 4:** Physiological parameters measured during incremental maximal exercise tests before (pre) and after (post) the 6‐week intervention.

	Paired samples test			Delta vs. delta	
Parameter, group	Pre	Post	*P*‐value; Cohen's *d* [95% CI]	Mean difference	*P*‐value, *q*‐value Cohen's *d* [95% CI]
${\dot{V}}_{O_{2}\mathrm{peak}}$, absolute (L min^−1^)					
Normoxia					
HWI	3.57 ± 0.49	4.05 ± 0.50	***P* < 0.01**; *d* = 0.97 [0.64, 1.72]	0.29 ± 0.39	*P* = 0.15, *q* = 0.24; *d* = 0.48 [−0.6, 1.43]
Control	3.58 ± 0.80	3.87 ± 0.91	***P* = 0.02**; *d* = 0.34 [0.14, 0.86]	0.48 ± 0.38	
Hypoxia					
HWI	2.54 ± 0.60	2.92 ± 0.65	***P* < 0.01**; *d* = 0.61 [0.31, 1.05]	0.38 ± 0.29	***P* = 0.04**, *q* = 0.10; *d* = 0.82 [−0.16, 1.6]
Control	2.38 ± 0.64	2.58 ± 0.71	***P* < 0.01**; *d* = 0.29 [0.20, 0.40]	0.20 ± 0.13	
${\dot{V}}_{O_{2}\mathrm{peak}}$, relative (mL kg^−1^ min^−1^)					
Normoxia					
HWI	47.7 ± 8.16	53.8 ± 6.12	***P* < 0.01**; *d* = 0.85 [0.32, 1.49]	6.10 ± 4.61	*P* = 0.23, *q* = 0.32; *d* = 0.42 [−0.64, 1.3]
Control	47.0 ± 9.76	51.5 ± 10.6	***P* < 0.01**; *d* = 0.41 [0.18, 1.04]	4.46 ± 4.92	
Hypoxia					
HWI	33.7 ± 7.11	38.6 ± 6.53	***P* < 0.01**; *d* = 0.72 [0.32, 1.28]	4.90 ± 3.67	*P* = 0.09, *q* = 0.17; *d* = 0.63 [−0.31, 1.5]
Control	31.5 ± 8.84	34.6 ± 9.68	***P* < 0.01**; *d* = 0.33 [0.23, 0.45]	3.11 ± 1.69	
First ventilatory threshold (V1 at power output)					
Normoxia					
HWI	194 ± 48.0	203 ± 42.2	*P* = 0.07; *d* = 0.21 [−0.06, 0.53]	9.50 ± 18.2	*P* = 0.38, *q* = 0.48; *d* = −0.13 [−1.02, 0.8]
Control	193 ± 40.4	205 ± 39.4	***P* = 0.04**; *d* = 0.29 [0.08, 0.55]	11.5 ± 10.0	
Hypoxia					
HWI	151 ± 21.9	166 ± 25.5	***P* < 0.01**; *d* = 0.63 [0.35, 1.08]	15.0 ± 12.9	*P* = 0.11, *q* = 0.19; *d* = 0.57 [−0.38, 1.5]
Control	163 ± 22.6	172 ± 23.1	***P* < 0.01**; *d* = 0.39 [0.19, 0.68]	9.00 ± 6.99	
Second ventilatory threshold (V2 at power output)					
Normoxia					
HWI	274 ± 44.2	287 ± 47.0	***P* < 0.01**; *d* = 0.29 [0.15, 0.61]	8.50 ± 9.14	*P* = 0.15, *q* = 0.24; *d* = 0.47 [−0.46, 1.3]
Control	276 ± 45.6	285 ± 43.0	***P* < 0.01**; *d* = 0.19 [0.05, 0.35]	13.5 ± 12.0	
Hypoxia					
HWI	215 ± 30.7	234 ± 34.1	***P* < 0.01**; *d* = 0.57 [0.21, 1.18]	18.5 ± 16.3	***P* = 0.05**, *q* = 0.11; *d* = 0.77 [−0.21, 1.5]
Control	221 ± 34.3	230 ± 36.5	***P* < 0.01**; *d* = 0.24 [0.06, 0.42]	8.50 ± 8.51	

*Note*: Data are presented as means ± SD. Parameters include relative and absolute ${\dot{V}}_{O_{2}\mathrm{peak}}$ and first and second ventilatory thresholds. Values in bold indicate a significant difference.

Across both groups, a weak positive correlation was observed between peak power output in hypoxia and haemoglobin concentration, whilst a moderate correlation was found between haemoglobin and ${\dot{V}}_{O_{2}\mathrm{peak}}$ (absolute and relative) in normoxia, but not in hypoxia. Additionally, haemoglobin concentration showed a moderate positive correlation with time‐to‐exhaustion trial performance (Table 5).

**TABLE 5:** Correlation analysis across groups between changes in haemoglobin concentration (post minus pre, delta) and changes in exercise performance outcomes in normoxic and hypoxic conditions.

Parameter	*P*	
Peak power output (W)		
Normoxia	0.18	*r* = 0.21
Hypoxia	**0.04**	*r* = 0.38
${\dot{V}}_{O_{2}\mathrm{peak}}$, absolute (L min^−1^)		
Normoxia	**0.01**	*rho* = 0.51
Hypoxia	0.11	*rho* = 0.28
${\dot{V}}_{O_{2}\mathrm{peak}}$, relative (mL kg^−1^ min^−1^)		
Normoxia	**0.01**	*rho* = 0.49
Hypoxia	0.17	*rho* = 0.22
TTE at 80% of ${\dot{V}}_{O_{2}\mathrm{peak}}$ (seconds)	**0.03**	*rho* = 0.41
TTE at 80% of ${\dot{V}}_{O_{2}\mathrm{peak}}$ (total power output)	**0.03**	*r* = 0.40

*Note*: Exercise performance metrics included peak power output, relative and absolute ${\dot{V}}_{O_{2}\mathrm{peak}}$, and time‐to‐exhaustion trial (TTE) results in seconds and total power output. Values in bold indicate a significant difference.

### 3.3 Physiological responses during the time‐to‐exhaustion trial in hypoxia

Examining physiological changes at different time points during the time‐to‐exhaustion trial, the post‐exercise HWI group exhibited higher $S_{pO_{2}}$ (1.71 ± 0.36 vs. 0.20 ± 1.79%; *P* = 0.01, *q* = 0.04; *d* = 1.17 [0.19, 2.3]) at 25% of the trial duration, and lower core temperature (−0.38 ± 0.19 vs. −0.06 ± 0.24°C; *P* < 0.01, *q* = 0.001; *d* = −1.48 [−2.15, −0.73]) at 50%, compared to the control group. By 75% of the trial, respiratory tidal volume was lower in the HWI group (−0.18 ± 0.21 vs. 0.16 ± 0.27 L; *P* < 0.01, *q* = 0.001; *d* = −1.43 [−2.13, −0.48]). No between‐group differences were observed in heart rate, ${\dot{V}}_{O_{2}}$, ${\dot{V}}_{E}$ and RER between 25% and 75% of the time‐to‐exhaustion trial (*P* and *q* > 0.05). At exhaustion (last 15 s), the HWI group presented higher absolute ${\dot{V}}_{O_{2}}$ (0.11 ± 0.73 vs. −0.19 ± 0.22 L min^−1^; *P* = 0.01, *q* = 0.04; *r*_rb_ = 0.65 [0.30, 0.85]), reduced tidal volume (−0.22 ± 0.43 vs. 0.24 ± 0.33 L; *P* = 0.01, *q* = 0.04; *d* = −1.20 [−1.86, −0.32]) and lower core temperature (−0.38 ± 0.21 vs. −0.04 ± 0.15°C; *P* < 0.01, *q* = 0.04; *d* = −1.86 [−2.59, 0.93]), whilst no changes were observed in heart rate, $S_{pO_{2}}$, RER or ${\dot{V}}_{E}$, (*P* and *q* > 0.05). The full statistical report is available in Supporting information, Table S1.

### 3.4 Physiological responses at 40% and 60% of ${\dot{V}}_{O_{2}\mathrm{peak}}$ in normoxic and hypoxic conditions

During the steady‐state exercise test at 40% of ${\dot{V}}_{O_{2}\mathrm{peak}}$ in normoxic conditions, although the post‐exercise HWI group demonstrated lower heart rate (−7.25 ± 6.54 vs. −1.92 ± 5.12 bpm; *d* = −0.91 [−1.79, 0.05]) and core temperature (−0.08 ± 0.13 vs. 0.04 ± 0.13°C; *d* = −0.90 [−1.58, −0.12]), these differences did not show statistical significance between groups (both *P* = 0.03, *q* = 0.08). Similarly, at 60% of ${\dot{V}}_{O_{2}\mathrm{peak}}$, a lower heart rate (−9.97 ± 9.16 vs. −3.35 ± 3.34 bpm; *d* = −0.96 [−1.91, −0.09]), core temperature (−0.14 ± 0.18 vs. 0.03 ± 0.16°C; *d* = −0.98 [−1.62, −0.12]) and respiratory tidal volume (−0.19 ± 0.18 vs. −0.05 ± 0.13 L; *d* = −0.89 [−2.2, −0.17]) were observed in the HWI group, but the between‐group comparison did not reach statistical significance (*P* = 0.02, *q* = 0.07; *P* = 0.02, *q* = 0.07; *P* = 0.03, *q* = 0.08; respectively). Additionally, no significant between‐group differences were found in RPE, $S_{pO_{2}}$, ${\dot{V}}_{O_{2}}$, ${\dot{V}}_{E}$ and RER at either 40% or 60% of ${\dot{V}}_{O_{2}\mathrm{peak}}$ (*P* and *q* > 0.05) (see Supporting information, Table S2).

At 40% of ${\dot{V}}_{O_{2}\mathrm{peak}}$ under hypoxic conditions, the HWI group presented lower values of heart rate (−9.33 ± 6.22 vs. −4.77 ± 3.60 bpm; *d* = −0.89 [−1.84, 0.26]) and core temperature (−0.22 ± 0.17 vs. 0.00 ± 0.13°C; *d* = −1.41[−2.31, −0.52]); however, only reduction in core temperature was statistically significant between groups (*P* = 0.03, *q* = 0.08 and *P* < 0.01, *q* = 0.001, respectively). At 60% of ${\dot{V}}_{O_{2}\mathrm{peak}}$, the HWI group also showed lower values of heart rate (−10.9 ± 8.13 vs. −4.67 ± 4.46 bpm; *d* = −0.95 [−1.84, 0.11]), core temperature (−0.26 ± 0.20 vs. −0.02 ± 0.15°C; *r*_rb_ = −0.58 [−0.85, −0.2]), ${\dot{V}}_{E}$ (−8.11 ± 5.40 vs. −3.45 ± 2.95 L min^−1^; *d* = −1.07 [−2.0, −0.15]) and respiratory tidal volume (−0.24 ± 0.31 vs. −0.01 ± 0.15 L; *d* = −0.89 [−1.76, 0.13]), as well as higher $S_{pO_{2}}$ (3.46 ± 1.32 vs. 1.25 ± 1.43%; *d* = 1.6 [0.65, 2.51]). However, between‐group statistical differences were only observed for core temperature and $S_{pO_{2}}$ (both *P* < 0.01, *q* = 001); no significant differences were found in heart rate, ${\dot{V}}_{E}$ (both *P* = 0.02, *q* = 0.07) or tidal volume (*P* = 0.03, *q* = 0.08). Additionally, no differences were observed in ${\dot{V}}_{O_{2}}$, RER or RPE at either 40% or 60% of ${\dot{V}}_{O_{2}\mathrm{peak}}$ in hypoxia (*P* and *q* > 0.05) (see Supporting information, Table S3).

## 4 DISCUSSION

This study provides novel evidence of environmental cross‐adaptation induced by a 6‐week post‐exercise HWI intervention. The HWI group exhibited greater improvements in time‐to‐exhaustion at 80% of ${\dot{V}}_{O_{2}\mathrm{peak}}$ under hypoxic conditions (13% $F_{iO_{2}}$: ∼4300 m simulated altitude) compared to control group. Furthermore, during the time‐to‐exhaustion trial, the HWI group demonstrated elevated $S_{pO_{2}}$ at 25%, lower core temperature at 50%, lower respiratory tidal volume at 75% and higher ${\dot{V}}_{O_{2}}$ and lower tidal volume in the final 15 s of the trial. Additionally, during hypoxic steady‐state exercise, lower core temperature was observed at 40% and 60% of ${\dot{V}}_{O_{2}\mathrm{peak}}$, along with elevated $S_{pO_{2}}$ at 60%. These findings suggest that post‐exercise HWI could serve as a feasible and accessible environmental stress stimulus, inducing a cross‐adaptation effect. This method may be particularly advantageous for those without access to hypoxic or heat chambers or those who cannot attend altitude camps.

That said, this study did not confirm the hypothesis that post‐exercise HWI would enhance maximal aerobic power under hypoxia and reduce physiological strain during steady‐state exercise in normoxia. However, these null findings should be interpreted cautiously, as several key measures were close to the significance threshold (α = 0.05), suggesting the possibility of Type II error due to limited sample size rather than definitive absence of effects. For instance, the peak power output in hypoxia (*P* = 0.03, *q* = 0.08; *d* = 0.86 [−0.13, 1.75]) suggests uncertainty rather than clear evidence against the effect, potentially indicating a false negative (Figure 3d). Similarly, physiological strain reductions in normoxic steady‐state exercise could be confirmed with a larger sample size, as indicated by lower heart rate (*P* = 0.03, *q* = 0.08; *d* = −0.91 [−1.79, 0.05]) and core temperature at 40% of ${\dot{V}}_{O_{2}\mathrm{peak}}$ (*P* = 0.03, *q* = 0.08; *d* = −0.90 [−1.58, −0.12]), and lower heart rate (*P* = 0.02, *q* = 0.07; *d* = −0.96 [−1.91, −0.09]), core temperature (*P* = 0.02, *q* = 0.07; *d* = −0.98 [−1.62, −0.12]) and respiratory tidal volume (*P* = 0.03, *q* = 0.08; *d* = −0.89 [−2.2, 0.17]) at 60 of ${\dot{V}}_{O_{2}\mathrm{peak}}$ (Supporting information, Table S2). Moreover, with greater statistical power, additional physiological changes in hypoxic conditions may have been confirmed, including decreased heart rate at 25% of the time‐to‐exhaustion (*P* = 0.02, *q* = 0.07; *d* = −1.19 [−2.55, 0.12]) and during the steady‐state exercise at 40% (*P* = 0.03, *q* = 0.08; *d* = −0.89 [−1.84, 0.26]) and 60% of ${\dot{V}}_{O_{2}\mathrm{peak}}$ (*P* = 0.02, *q* = 0.07; *d* = −0.95 [−1.84, 0.11]), increased $S_{pO_{2}}$ at 50% of the time‐to‐exhaustion (*P* = 0.02, *q* = 0.07; *d* = 1.03 [0.15, 1.86]) and decreased ${\dot{V}}_{E}$ (*P* = 0.02, *q* = 0.07; *d* = −1.07 [−2.0, −0.15]) and tidal volume at 60% of ${\dot{V}}_{O_{2}\mathrm{peak}}$ (*P* = 0.03, *q* = 0.08; *d* = −0.89 [−1.76, 0.13]) (Supporting information, Tables S1 and S3). Therefore, future studies with a greater sample size are warranted to confirm these potential improvements.

### 4.1 Post‐exercise HWI as a heat acclimation and cross‐adaptation strategy

The effectiveness of the heat acclimatisation induced by post‐exercise HWI is supported by reductions in resting core temperature (−0.22 ± 0.12°C, mean ± SD) and decreases in core temperature after both the HIIT session (from 38.1 ± 0.2°C to 37.8 ± 0.3°C) and the 42°C HWI session (from 39.2 ± 0.2°C to 38.5 ± 0.4°C) following the 6‐week intervention (Figure 2). These findings confirm previous research highlighting that post‐exercise HWI is a feasible alternative for exercise heat acclimation (McIntyre et al., 2021; Zurawlew et al., 2016). However, this study did not confirm that post‐exercise HWI strategy decreases physiological strain during submaximal exercise intensity in thermoneutral and normoxic environments as previously reported (McIntyre et al., 2021; Philp et al., 2022; Zurawlew et al., 2019, 2016). Although some physiological responses, such as lower heart rate, core temperature and respiratory tidal volume, suggest a potential reduction in strain during steady‐state exercise in normoxia, a larger sample size is needed to confirm these effects. Beyond thermal stress adaptation, this study detected some cross‐adaptation effects, with reductions in ${\dot{V}}_{E}$ and increases in $S_{pO_{2}}$ observed during steady‐state exercise at 60% of ${\dot{V}}_{O_{2}\mathrm{peak}}$ in hypoxia, but not in normoxia. Additionally, the HWI group presented higher absolute ${\dot{V}}_{O_{2}}$ (*P* = 0.01, *q* = 0.04; *r*_rb_ = 0.65 [0.30, 0.85]) alongside lower tidal volume (*P* = 0.01, *q* = 0.04; *d* = −1.2 [−1.86, −0.32]) at exhaustion (last 15 s) at 80% of ${\dot{V}}_{O_{2}\mathrm{peak}}$ under hypoxia, suggesting improved respiratory efficiency (Supporting information, Table S1).

Interestingly, haemoglobin concentration showed positive correlations with ${\dot{V}}_{O_{2}\mathrm{peak}}$ in normoxia (absolute and relative), but not in hypoxia. Conversely, haemoglobin concentration was positively correlated with peak power output in hypoxia but not in normoxia (Table 5). Lundby et al. (2023) reported increases in haemoglobin mass after 5 weeks of exercise heat acclimation (50‐min sessions, five times a week, using thin wool clothing and nylon rain jacket to reduce heat dissipation and increase body heat storage), and found it positively correlated with elevated ${\dot{V}}_{O_{2}\mathrm{peak}}$ in normoxia (*P* < 0.01; *r* = 0.44). Additionally, they observed positive correlations between haemoglobin mass and enhanced exercise performance, including power output at 3 mmol L^−1^ [lactate] (*P* < 0.01; *r* = 0.5) and average power during a 15‐min all‐out cycling test (*P* < 0.01; *r* = 0.49). Similarly, Cubel et al. (2024) observed increased total haemoglobin mass along with increased peak power output during the incremental test following a 5‐week exercise heat acclimation intervention (60‐min sessions, six times per week, home‐based training using winter clothing to keep core temperature at 38.5°C); however, no changes were observed in ${\dot{V}}_{O_{2}\mathrm{peak}}$ in normoxia (correlation test was not completed). Although these studies (Cubel et al., 2024; Lundby et al., 2023) did not assess the effects of heat acclimation on acute hypoxia, their findings suggest that longer interventions may be required to increase haemoglobin mass and optimise exercise performance. In line with this, shorter heat acclimation interventions (up to 10 sessions) often fail to induce similar changes (Gibson et al., 2015; Lee et al., 2016; McIntyre et al., 2021; Zurawlew et al., 2019).

Plasma volume expansion may also be an important mechanism decreasing physiological strain during exercise, as it can reduce heart rate due to increased ventricular preload, leading to cardiovascular stability (Convertino, 1991, 2007) and decrease core temperature due to increased sweating and heat‐release balance pathways (Périard et al., 2015). Although the benefits of plasma volume expansion from exercise heat acclimatisation are well‐documented, most studies have used short‐term (≤7 days) or medium‐term (8–14 days) interventions (see Tyler et al. (2024) for a systematic review and meta‐analysis). It has been suggested that after prolonged exercise heat acclimation, increases in haemoglobin mass are associated with the rate of plasma expansion (*P* = 0.02, *r* = 0.49) (Oberholzer et al., 2019); however, the impact of longer heat acclimation interventions (e.g., 5 weeks) on plasma volume remain unclear. Although Lundby et al. (2023) found an increase in plasma volume along with increased haemoglobin mass, Cubel et al. (2024) found an increase in haemoglobin mass but not plasma volume compared to the control condition, consistent with the present study findings. The absence of plasma volume expansion after the 6‐week post‐exercise HWI intervention may have contributed to the non‐significant reductions in heart rate during exercise under normoxic and hypoxic conditions.

Racinais et al. (2024) examined the haematological effects of a 3‐week exercise heat acclimation intervention and found that plasma volume increased after 11 days but returned to baseline levels after 3 weeks; meanwhile, haemoglobin mass initially decreased after 4 days but returned to baseline by the end of the intervention. Therefore, although the effects of prolonged heat acclimation on plasma volume expansion remain unclear, the present findings, along with previous research (Cubel et al., 2024; Lundby et al., 2023; Oberholzer et al., 2019), suggest that longer heat acclimation interventions may provide enhanced cross‐adaptation benefits, particularly by increasing haemoglobin concentration.

### 4.2 Post‐exercise HWI and physiological strain cross‐adaptations

Regarding cardiorespiratory responses from cross‐adaptation interventions, the debate continues concerning its results. Although some studies have reported reductions in ${\dot{V}}_{O_{2}}$ (Salgado et al., 2020; Sotiridis et al., 2019), RER, ${\dot{V}}_{E}$ (Sotiridis et al., 2019) and heart rate (Gibson et al., 2015; Sotiridis et al., 2019) as well as increased oxygen saturation (Gibson et al., 2015; Lee et al., 2016) during submaximal exercise under hypoxia, other studies have not observed these changes. For example, some studies found no differences in ${\dot{V}}_{O_{2}}$ (Lee et al., 2016; White et al., 2015), RER (Gibson et al., 2015; White et al., 2015), ${\dot{V}}_{E}$ (Gibson et al., 2015; White et al., 2015), heart rate (Lee et al., 2016; White et al., 2015) or oxygen saturation (Heled et al., 2012; White et al., 2015). These discrepancies may stem from variations in study design, such as differences in exercise session duration (60–120 min), heat acclimation protocol length (3–10 sessions), hypoxic protocol ($F_{iO_{2}}$ ranging from 0.12 to 0.16) and whether or not hyperbaric pressure was used (normobaric or 86–114 mmHg). Additionally, hypoxic exercise performance testing methods vary, with maximal tests ranging from time trials to incremental tests and lactate accumulation onset (see Willmott et al. (2024) for a systematic review). Nevertheless, meta‐analyses suggest that cross‐acclimation has no effect on ${\dot{V}}_{O_{2}}$, RER or ${\dot{V}}_{E}$ but shows a small effect on oxygen saturation and a moderate effect on reducing heart rate (Willmott et al., 2024).

In the present study, no differences in ${\dot{V}}_{O_{2}}$, RER or heart rate were observed between groups during steady‐state exercise at 40% and 60% of ${\dot{V}}_{O_{2}\mathrm{peak}}$ under normoxia and hypoxia, as well as during 25–75% of the time‐to‐exhaustion trial in hypoxia. Nevertheless, the HWI group exhibited lower respiratory tidal volume and ${\dot{V}}_{E}$, along with increased $S_{pO_{2}}$ under hypoxia (both within and between groups), suggesting that prolonged heat acclimation through post‐exercise HWI may enhance respiratory efficiency in hypoxia. The HWI group was able to maintain target exercise intensity with ${\dot{V}}_{O_{2}}$ similar to the control group, but with lower tidal volume and ${\dot{V}}_{E}$, and elevated oxygen saturation, indicating lower respiratory strain during acute hypoxia. However, these changes were insufficient to induce decreases in perceptual responses. Although both groups reported decreased RPE following the 6‐week intervention, the reductions in respiratory and thermal strain did not lead to notable differences in RPE between groups. This aligns with findings from Lee et al. (2016) but contrasts with Sotiridis et al. (2019), who observed reduced perceptual strain following cross‐acclimation.

### 4.3 Post‐exercise HWI and exercise performance in acute hypoxia

The primary outcomes of this study were the exercise performance tests under hypoxic conditions, assessed through the aerobic peak power test and time‐to‐exhaustion trial. Despite using different maximal exercise test protocols in hypoxia, meta‐analysis revealed that the cross‐adaptation interventions (up to 10 sessions) have a small effect on increasing peak power output and improving time trial duration (Willmott et al., 2024). Following 6 weeks of post‐exercise HWI, the present findings suggest a large effect on enhanced peak power output (*d* = 0.86 [−0.13, 1.75]), although this increase was not statistically significant (*P* = 0.03, *q *= 0.08; Figure 3d). The time‐to‐exhaustion trial also indicated a large effect, with significant improvements in both duration (10.4%; *d* = 1.23 [0.15, 2.01]) and power output (10.9%; *d* = 1.35 [0.28, 2.25]; *P* and *q* < 0.01; Figure 4b,d), suggesting that longer heat acclimation interventions may be more effective in enhancing exercise performance in hypoxia.

Although this study did not detect changes in ${\dot{V}}_{O_{2}\mathrm{peak}}$ between groups, previous research has shown that improvements in peak power can occur without significant changes in ${\dot{V}}_{O_{2}\mathrm{peak}}$ following a 5‐week heat acclimation (Cubel et al., 2024). The cross‐adaptation effect on hypoxic exercise performance may be linked to physiological threshold modifications, as evidenced by Heled et al. (2012), who reported a delayed onset of blood lactate accumulation under hypoxia (15.6% $F_{iO_{2}}$) after 12 days of heat acclimation. However, this study did not find any changes in the first or second physiological thresholds under normoxic or hypoxic conditions, as determined by ventilatory thresholds (Table 4.) The improvements in time‐to‐exhaustion may be partially attributed to reductions in core temperature and respiratory tidal volume, and increased $S_{pO_{2}}$ throughout the trial (indicating improved breathing efficiency), alongside elevated absolute ${\dot{V}}_{O_{2}}$ at the end of the performance test. Moreover, the enhanced peak power output and time‐to‐exhaustion may also be partially explained by their positive correlations with increased haemoglobin concentration (Table 5). Therefore, the improvements in exercise performance in hypoxia following a post‐exercise HWI intervention may result from various mechanisms, including reductions in thermal and respiratory strain and elevated levels of haemoglobin concentration.

### 4.4 Limitations

The present study measured haemoglobin concentration but no haemoglobin mass, limiting the comparison with previous studies that did (Cubel et al., 2024; Lundby et al., 2023). Furthermore, plasma volume was estimated using the Dill and Costill equation based on haematocrit and haemoglobin concentrations rather than being directly measured, reducing accuracy compared to previous studies. Another noteworthy limitation is the exercise training design. Although participants were orientated to maintain their regular physical activities on non‐supervised HIIT days and avoid starting new activities or altering training intensities and volumes during the intervention, the assessment of their weekly activity volume was self‐reported and not objectively measured. Moreover, despite applying a false discovery rate analysis to reduce the risk of Type I error, the small sample size in this study increases the likelihood risk of Type II errors. Lastly, this study explored the potential benefits of heat acclimation on exercise performance in acute normobaric–hypoxic conditions, and thus caution should be considered when extrapolating these adaptations to high‐altitude settings. Future studies should investigate the effects of post‐exercise HWI in actual high‐altitude environments.

### 4.5 Conclusion

Six weeks of post‐exercise HWI intervention resulted in greater improvements in time‐to‐exhaustion at 80% of ${\dot{V}}_{O_{2}\mathrm{peak}}$ in acute hypoxia (13% $F_{iO_{2}}$) compared to exercise training alone. This enhancement in normobaric–hypoxic exercise performance is potentially explained by increased haemoglobin concentration, lower core temperature and improved respiratory efficiency (i.e., lower tidal volume and ${\dot{V}}_{E}$, along with increased absolute ${\dot{V}}_{O_{2}}$ and oxygen saturation). Additionally, post‐exercise HWI intervention may further enhance aerobic peak power in hypoxia and reduce physiological strain during steady‐state exercise under both normoxic and hypoxic conditions (i.e., lower core temperature, heart rate, respiratory tidal volume and ${\dot{V}}_{E}$); however, further studies with a larger sample size are needed to confirm these findings.

Hot‐water baths offer a practical and accessible heat acclimation strategy for athletes ranging from recreational to elite levels, while providing minimal interference to the training regimen. This intervention is particularly suitable for training camps and travel scenarios. Post‐exercise HWI emerges as an effective, low‐coast and accessible method for heat acclimation and has the potential to improve exercise performance at high altitudes. Importantly, the HWI sessions in this study were conducted at 42°C for 40–50 min to the chest level, resulting in a core temperature of 39.2 ± 0.2°C in the first week of intervention. Lower thermal stress sessions may not induce the same results observed in this study.

## AUTHOR CONTRIBUTIONS

P.R., J.S.L. and A.H. were responsible for the study's conception and design. P.R., L.L.S., J.S.L. and H.S.L. conducted the study intervention and data acquisition. P.R., L.L.S. and H.S.L. performed the data analysis and interpretation. J.S.L. and A.H. supervised the study. P.R. drafted the manuscript, and all authors reviewed and contributed to the final version. All authors have read and approved the final version of this manuscript and agree to be accountable for all aspects of the work in ensuring that questions related to the accuracy or integrity of any part of the work are appropriately investigated and resolved. All persons designated as authors qualify for authorship, and all those who qualify for authorship are listed.

## CONFLICT OF INTEREST

None declared.

## FUNDING INFORMATION

None.

## Supporting information



Tables S1–S3.

## Data Availability

All data for the main outcomes of this study are plotted in the figures as paired individual data and change scores. Data for the secondary outcomes are available from the corresponding author upon reasonable request.
