# Supplementary material for: Heat to hypoxia cross‐adaptation: Effects of 6‐week post‐exercise hot‐water immersion on exercise performance in acute hypoxia
Source: Exp Physiol. 2025 May 11;111(1):124–38. doi: 10.1113/EP092726 (PMC12756880; doi:10.1113/EP092726)
Supplement: Supplementary file 1 — Tables S1–S3. [file EPH-111-124-s001.docx]

Table S1 - Physiological responses during the time-to-exhaustion at 80% of V̇O_2peak_ under hypoxia before (Pre) and after (Post) the six-week intervention. Parameters measured include heart rate, peripheral oxygen saturation (SpO_2_), oxygen consumption (V̇O_2_), respiratory exchange ratio (RER, V̇CO_2_/V̇O_2_), ventilation (V̇_E_), respiratory tidal volume and core temperature (rectal). Data are presented as mean ± SD.

|  |  | **Paired samples test** | | |  | **Delta vs. delta** | |
| --- | --- | --- | --- | --- | --- | --- | --- |
| **Parameter** | **Group** | **Pre**  **(mean ± SD)** | **Post**  **(mean ± SD)** | **p-value; Cohen’s *d* [95% CI]** |  | **Mean difference**  **(mean ± SD)** | **p-value, q-value**  **Cohen’s *d* [95% CI]** |
| **At 25% of the TTE** |  | | | | | | |
| Heart rate  (BPM) | HWI  Control | 162 ± 11.9  164 ± 5.88 | 159 ± 10.6  164 ± 7.25 | **p =.04**; *d* =-0.32 [-1.25, 0.61]  p =.35; *r*_rb_ =0.01 [-0.45, 0.48] |  | -3.66 ± 5.95  -0.05 ± 2.92 | **p =.05**, q =.11; *d* =-0.77 [-1.45, -0.27] |
| SpO_2_  (%) | HWI  Control | 77.8 ± 2.68  72.1 ± 5.77 | 79.5 ± 2.75  72.3 ± 5.43 | **p <.01**; *d* =0.63 [0.49, 0.87]  p =.36; *d* =0.03 [-0.17, 0.36] |  | 1.71 ± 0.36  0.20 ± 1.79 | **p =.01, q =**.**04**; *d* =1.17 [0.19, 2.3] |
| V̇O_2_  (L.min^-1^) | HWI  Control | 2.16 ± 0.52  2.27 ± 0.32 | 2.24 ± 2.52  2.19 ± 0.43 | p =.39; *d* =0.16 [-0.16, 0.64]  p =.17; *d* =-0.23 [-0.72, 0.05] |  | 0.08 ± 0.28  -0.08 ± 0.18 | p =.14, q =.23; *d* =0.69 [-0.24, 1.51] |
| RER  (ratio) | HWI  Control | 1.17 ± 0.29  1.21 ± 0.20 | 1.26 ± 0.19  1.15 ± 0.20 | p =.34; *d* =0.39 [-0.59, 1.1]  p =.10; *d* =0.65 [-0.01, 1.46] |  | 0.09 ± 0.30  0.13 ± 0.22 | p =.78, q =.85; *d* =-0.12 [-1.1, 0.81] |
| V̇_E_  (L.min^-1^) | HWI  Control | 76.9 ± 10.3  71.0 ± 17.2 | 79.2 ± 7.93  72.7 ± 17.3 | p =.48; *d* =0.25 [-0.52, 0.96]  p =.46; *d* =0.09 [-0.15, 0.41] |  | 1.66 ± 6.82  2.36 ± 10.0 | p =.86, q =.88; *d* =0.08 [-0.87, 1.01] |
| Tidal volume  (L) | HWI  Control | 2.60 ± 0.63  2.62 ± 0.22 | 2.62 ± 0.58  2.80 ± 0.59 | p =.57; *d* =0.02 [-0.10, 0.10]  **p =.01**; *d* =0.31 [0.07, 0.49] |  | 0.02 ± 0.09  0.18 ± 0.17 | **p =.02**, q =.07; *d* =-1.19 [-2.55, 0.12] |
| **At 50% of the TTE** |  | | | | | | |
| Heart rate  (BPM) | HWI  Control | 170 ± 11.2  172 ± 5.00 | 165 ± 11.4  170 ± 6.03 | **p =.02**; *d* =-0.39 [-0.72, -0.07]  **p =.05;** *r*_rb_ =0.33 [-0.15, 0.70] |  | -4.47 ± 5.83  -2.32 ± 3.40 | p =.17, q =.25; *d* =-0.45 [-1.39, 0.51] |
| SpO_2_  (%) | HWI  Control | 75.3 ± 4.00  71.1 ± 6.32 | 77.6 ± 3.16  71.2 ± 5.73 | **p <.01**; *d* =0.64 [0.31, 1.18]  p =.48; *d* =0.01 [-0.23, 0.29] |  | 2.32 ± 2.42  0.03 ± 2.01 | **p =.02**, q =.07; *d* =1.03 [0.15, 1.86] |
| V̇O_2_  (L.min^-1^) | HWI  Control | 2.40 ± 0.65  2.35 ± 0.29 | 2.47 ± 0.64  2.21 ± 0.37 | p =.56; *d* =0.10 [-0.25, 0.47]  **p =.04**; *d* =-0.44 [-0.98, -0.12] |  | 0.07 ± 0.38  -0.15 ± 0.19 | p =.13, q =.22; *d* =0.71 [-0.33, 1.48] |
| RER  (ratio) | HWI  Control | 1.27 ± 0.31  1.20 ± 0.12 | 1.24 ± 0.26  1.31 ± 0.16 | p =.67; *d* =-0.11 [-0.79, 0.34]  p =.13; *d* =0.78 [-0.21, 1.74] |  | -0.03 ± 0.23  0.11 ± 0.21 | p =.16, q =0.25; *d* =-0.65 [-1.45, 0.32] |
| V̇_E_  (L.min^-1^) | HWI  Control | 89.5 ± 11.5  84.0 ± 19.5 | 90.3 ± 6.84  84.5 ± 17.7 | p =.75; *d* =0.09 [-0.59, 0.67]  p =.84; *d* =0.02 [-0.26, 0.33] |  | 0.85 ± 8.07  0.50 ± 7.40 | p =.92, q =.92; *d* =0.05 [-0.89, 1.0] |
| Tidal volume  (L) | HWI  Control | 2.65 ± 0.56  2.68 ± 0.49 | 2.71 ± 0.59  2.83 ± 0.54 | p =.18; *d* =0.11 [-0.01, 0.37]  p =.07; *d* =0.29 [-0.01, 0.65] |  | 0.07 ± 0.14  0.15 ± 0.23 | p =.34, q =.44; *d* =-0.44 [-1.48, 0.54] |
| Rectal temperature (ºC) | HWI  Control | 38.0 ± 0.38  37.8 ± 0.28 | 37.6 ± 0.29  37.7 ± 0.27 | **p <.01**; *d* =-1.11 [-1.77, -0.66]  p =.34; *d* =-0.22 [-0.57, 0.36] |  | -0.38 ± 0.19  -0.06 ± 0.24 | **p<.01, q =.001**; *d* =-1.48[-2.15, -0.73] |
| **At 75% of the TTE** |  | | | | | | |
| Heart rate  (BPM) | HWI  Control | 175 ± 12.0  178 ± 4.31 | 170 ± 10.4  176 ± 5.87 | **p =.02**; *d* =-0.42 [-0.87, -0.06]  p =.15; *r*_rb_ =0.16 [-0.30, 0.55] |  | -4.74 ± 6.60  -1.38 ± 3.46 | p =.08, q =.15; *d* =-0.64 [-1.45, 0.23] |
| SpO_2_  (%) | HWI  Control | 73.3 ± 5.20  69.7 ± 70.4 | 75.8 ± 3.28  70.4 ± 5.11 | **p =.01**; *r*_rb_ =-0.28 [-0.65, 0.15]  p =.33; *d* =0.11 [-0.46, 0.60] |  | 2.51 ± 2.31  0.62 ± 4.42 | p =.89, q =.90; *d* =0.49 [-0.52, 1.3] |
| V̇O_2_  (L.min^-1^) | HWI  Control | 2.55 ± 0.60  2.47 ± 0.37 | 2.63 ± 0.69  2.35 ± 0.52 | p =.51; *d* =0.13 [-0.37, 0.45]  p =.08; *r*_rb_ =0.32 [-0.10, 0.65] |  | 0.09 ± 0.39  -0.12 ± 0.32 | p =.22, q =.31; *d* =0.57 [-0.52, 1.79] |
| RER  (ratio) | HWI  Control | 1.29 ± 0.28  1.22 ± 0.14 | 1.26 ± 0.25  1.29 ± 0.21 | p =.74; *d* =-0.11 [-0.79, 0.48]  p =.35; *d* =0.42 [-0.59, 1.38] |  | -0.03 ± 0.26  0.07 ± 0.25 | p =.36, q =.46; *d* =-0.41 [-1.41, 0.57] |
| V̇_E_  (L.min^-1^) | HWI  Control | 99.9 ± 12.5  100 ± 24.9 | 101 ± 6.07  100 ± 24.0 | p =.84; *d* =0.07 [-0.66, 0.97]  p =.97; *d* =-0.01[-0.28, 0.14] |  | 1.66 ± 6.82  2.36 ± 10.0 | p =.86, q =.88; *d* =0.08 [-0.91, 1.02] |
| Tidal volume  (L) | HWI  Control | 2.79 ± 0.75  2.70 ± 0.66 | 2.61 ± 0.64  2.86 ± 0.64 | **p =.02**; *d* =-0.26 [-0.54, -0.07]  p =.09; *d* =0.24 [0.01, 0.71] |  | -0.18 ± 0.21  0.16 ± 0.27 | **p<.01, q =.001**; *d* =-1.43[-2.13, -0.48] |
| **At 100% of the TTE** |  | | | | | | |
| Heart rate  (BPM) | HWI  Control | 177 ± 12.2  180 ± 5.59 | 174 ± 10.9  179 ± 6.34 | **p =.05**; *d* =-0.29 [-0.75, 0.01]  p =.22; *d* =-0.19 [-0.64, 0.33] |  | -3.47 ± 5.99  -1.16 ± 4.55 | p =.17, q =.25; *d* =-0.43 [-1.31, 0.52] |
| SpO_2_  (%) | HWI  Control | 77.0 ± 7.25  67.9 ± 5.30 | 74.5 ± 5.43  68.3 ± 3.74 | **p =.02**; *d* =0.40 [-0.01, 0.74]  p =.34; *d* =0.08 [-0.51, 0.45] |  | 2.57 ± 3.60  0.41 ± 3.11 | p =.08, q =.15; *d* =0.64 [-0.29, 1.48] |
| V̇O_2_  (L.min^-1^) | HWI  Control | 2.57 ± 0.67  2.77 ± 0.41 | 2.68 ± 0.76  2.57 ± 0.52 | p =.64; *d* =0.15 [-0.75, 0.73]  **p =.02**; *d* =-0.42 [-0.88, -0.14] |  | 0.11 ± 0.73  -0.19 ± 0.22 | **p =.01, q =.04**; *r*_rb_ =0.65 [0.30, 0.85] |
| RER  (ratio) | HWI  Control | 1.34 ± 0.20  1.21 ± 0.16 | 1.27 ± 0.26  1.27 ± 0.19 | p =.33; *d* =-0.23 [-0.79, 0.49]  p =.47; *d* =0.32 [-0.64, 1.15] |  | -0.07 ± 0.21  0.06 ± 0.24 | p =.23, q =.32; *d* =-0.56 [-1.64, 0.49] |
| V̇_E_  (L.min^-1^) | HWI  Control | 105 ± 13.3  113 ± 33.2 | 108 ± 7.87  113 ± 31.8 | p =.24; *d* =0.29 [-0.31, 0.79]  p =.82; *d* =-0.01 [-0.32, 0.16] |  | 3.20 ± 8.01  -0.60 ± 8.43 | p =.31, q =.41; *d* =0.46 [-0.52, 1.37] |
| Tidal volume  (L) | HWI  Control | 2.68 ± 0.81  2.65 ± 0.72 | 2.46 ± 0.66  2.89 ± 0.77 | p =.16; *r*_rb_ =-0.12 [-0.50, 0.30]  **p =.04**; *d* =0.32 [0.01, 0.76] |  | -0.22 ± 0.43  0.24 ± 0.33 | **p =.01, q =.04**; *d* =-1.2 [-1.86, -0.32] |
| Rectal temperature (ºC) | HWI  Control | 38.2 ± 0.35  37.9 ± 0.27 | 37.8 ± 0.23  37.8 ± 0.29 | **p <.01**; *d* =-1.28 [-1.95, -0.77]  p =.42; *d* =-0.14 [-0.41, 0.28] |  | -0.38 ± 0.21  -0.04 ± 0.15 | **p <.01, q =.001**; *d* =-1.86 [-2.59, 0.93] |

Table S2 - Physiological responses during the steady-state exercise test at 40% and 60% of the V̇O_2peak_ under normoxia before (Pre) and after (Post) the six-week intervention. Parameters measured include rate of perceived exertion (RPE), core temperature (rectal), heart rate, peripheral oxygen saturation (SpO_2_), oxygen consumption (V̇O_2_), respiratory exchange ratio (RER, V̇CO_2_/V̇O_2_), ventilation (V̇_E_), and respiratory tidal volume. Data are presented as mean ± SD.

|  |  | **Paired samples test** | | |  | **Delta vs. delta** | |
| --- | --- | --- | --- | --- | --- | --- | --- |
| **Parameter** | **Group** | **Pre**  **(mean ± SD)** | **Post**  **(mean ± SD)** | **p-value; Cohen’s *d* [95% CI]** |  | **Mean difference**  **(mean ± SD)** | **p-value, q-value**  **Cohen’s *d* [95% CI]** |
| **At 40% of VO_2max_** |  | | | | | | |
| RPE  (A.U.) | HWI  Control | 9.10 ± 1.10  10.4 ± 1.35 | 8.70 ± 1.06  9.80 ± 1.32 | p =.19; *d* =-0.37 [-1.24, 0.32]  p =.09; *d* =-0.45 [-1.2, 0.23] |  | -0.40 ± 1.35  -0.61 ± 1.35 | p =.37, q =.47; *d* =0.16 [-0.77, 1.18] |
| Rectal temperature (ºC) | HWI  Control | 37.3 ± 0.27  37.2 ± 0.32 | 37.2 ± 0.22  37.2 ± 0.25 | **p =.05**; *r*_rb_ =0.18 [-0.25, 0.55]  p =.82; *d* =0.14 [-0.18, 0.49] |  | -0.08 ± 0.13  0.04 ± 0.13 | **p =.03**, q =.08; *d* =-0.90 [-1.58, -0.12] |
| Heart rate  (BPM) | HWI  Control | 127 ± 7.18  129 ± 11.4 | 119 ± 9.34  127 ± 11.9 | **p <.01**; *d* =-0.87 [-1.98, -0.33]  p =.13; *d* =-0.16 [-0.51, 0.10] |  | -7.25 ± 6.54  -1.92 ± 5.12 | **p =.03**, q =.08; *d* =-0.91[-1.79, 0.05] |
| SpO_2_  (%) | HWI  Control | 96.2 ± 2.90  97.0 ± 1.30 | 96.4 ± 2.08  97.1 ± 0.91 | p =.34; *d* =0.07 [-0.31, 0.47]  p =.44; *d* =0.05 [-0.69, 0.95] |  | 0.19 ± 1.44  0.06 ± 1.33 | p =.42, q =.51; *d* =0.09 [-0.92, 0.98] |
| V̇O_2_  (L.min^-1^) | HWI  Control | 1.93 ± 0.26  2.03 ± 0.38 | 1.84 ± 0.27  1.92 ± 0.26 | **p =.03**; *d* =-0.34 [-0.69, -0.07]  p =.08; *d* =-0.31 [-0.63, 0.25] |  | -0.09 ± 0.13  -0.11 ± 0.19 | p =.62, q =.71; *d* =0.14 [-0.83, 1.11] |
| RER  (ratio) | HWI  Control | 0.87 ± 0.03  0.87 ± 0.07 | 0.87 ± 0.04  0.86 ± 0.04 | p =.81; *d* =0.32 [-0.44, 0.92]  p =.35; *d* =-0.13 [-0.83, 0.59] |  | 0.01 ± 0.03  0.01 ± 0.06 | p =.79, q =.85; *d* =0.37 [-0.66, 1.32] |
| V̇_E_  (L.min^-1^) | HWI  Control | 41.3 ± 2.41  41.0 ± 6.05 | 38.1 ± 2.41  39.5 ± 5.24 | **p <.01**; *d* =-0.92 [-1.58, -0.37]  p =.11; *d* =-0.27 [-0.76, 0.19] |  | -3.21 ± 2.75  -1.53 ± 3.71 | p =.13, q =.22; *d* =-0.51 [-1.4, 0.45] |
| Tidal volume  (L) | HWI  Control | 2.03 ± 0.53  2.14 ± 0.54 | 1.86 ± 0.45  2.12 ± 0.50 | **p =.02**; *d* =0.34 [0.07, 0.67]  p =.39; *d* =0.03 [-0.28, 0.31] |  | -0.17 ± 0.23  -0.02 ± 0.22 | p =.08, q =.15; *d* =-0.66 [-1.55, -0.29] |
| **At 60% of VO_2max_** |  | | | | | | |
| RPE  (A.U.) | HWI  Control | 12.4 ± 1.35  13.3 ± 0.95 | 11.0 ± 1.89  12.3 ± 1.06 | **p <.01**; *d* -0.85 [-1.59, -0.40]  p =.99; *r*_rb_ =0.49 [0.10, 0.75] |  | -1.40 ± 0.82  -1.00 ± 0.82 | p =.22, q =.31; *d* =-0.36 [-1.29, 0.59] |
| Rectal temperature (ºC) | HWI  Control | 37.5 ± 0.31  37.4 ± 0.25 | 37.4 ± 0.21  37.4 ± 0.17 | **p =.02**; *d* =-0.52 [-1.16, -0.14]  p =.71; *d* =0.14 [-0.32, 0.74] |  | -0.14 ± 0.18  0.03 ± 0.16 | **p =.02**, q =.07; *d* =-0.98 [-1.62, -0.12] |
| Heart rate  (BPM) | HWI  Control | 151 ± 10.4  153 ± 12.2 | 141 ± 7.84  150 ± 13.4 | **p <.01**; *d* =-1.08 [-1.99, -0.39]  **p <.01**; *d* =-0.26 [-0.54, -0.06] |  | -9.97 ± 9.16  -3.35 ± 3.34 | **p =.02**, q =.07; *d* =-0.96 [-1.91, -0.09] |
| SpO_2_  (%) | HWI  Control | 95.2 ± 2.41  95.5 ± 1.35 | 95.4 ± 2.05  95.7 ± 0.96 | p =.30; *d* =0.11 [-0.49, 0.45]  p =.35; *d* =0.09 [-0.43, 0.77] |  | 0.25 ± 1.45  0.11 ± 0.91 | p =.40, q =.49; *d* =0.11 [-0.86, 1.01] |
| V̇O_2_  (L.min^-1^) | HWI  Control | 2.60 ± 0.48  2.71 ± 0.44 | 2.54 ± 0.40  2.61 ± 0.42 | p =.16; *d* =-0.14 [-0.35, 0.14]  p =.06; *d* =-0.21 [-0.44, 0.04] |  | -0.06 ± 0.19  -0.09 ± 0.16 | p =.64, q =.72; *d* =0.17 [-0.79, 1.15] |
| RER  (ratio) | HWI  Control | 0.94 ± 0.04  0.95 ± 0.06 | 0.94 ± 0.04  0.92 ± 0.05 | p =.35; *d* =-0.07 [-0.44, 0.29]  p =.11; *d* =-0.44 [-1.31, 0.22] |  | 0.00 ± 0.02  -0.03 ± 0.06 | p =.84, q =.88; *d* =0.47 [-0.59, 1.39] |
| V̇_E_  (L.min^-1^) | HWI  Control | 58.6 ± 7.73  57.4 ± 7.59 | 54.5 ± 7.35  56.2 ± 6.28 | **p =.02**; *d* =-0.54 [-1.11, -0.15]  p =.09; *d* =-0.18 [-0.45, 0.08] |  | -4.07 ± 5.22  -1.26 ± 2.75 | p =.07, q =.15; *d* =-0.67 [-1.56, 0.31] |
| Tidal volume  (L) | HWI  Control | 2.45 ± 0.58  2.52 ± 0.58 | 2.26 ± 0.51  2.47 ± 0.56 | **p <.01**; *d* =0.35 [0.10, 0.61]  p =.12; *d* =0.09 [-0.04, 0.29] |  | -0.19 ± 0.18  -0.05 ± 0.13 | **p =.03**, q =.08; *d* =-0.89 [-2.2, -0.17] |

Table S3 - Physiological responses during the steady-state exercise test at 40% and 60% of the V̇O_2 max_ under hypoxia before (Pre) and after (Post) the six-week intervention. Parameters measured include rate of perceived exertion (RPE), core temperature (rectal), heart rate, peripheral oxygen saturation (SpO_2_), oxygen consumption (V̇O_2_), respiratory exchange ratio (RER, V̇CO_2_/V̇O_2_), ventilation (V̇_E_), and respiratory tidal volume. Data are presented as mean ± SD.

|  |  | **Paired samples test** | | |  | **Delta vs. delta** | |
| --- | --- | --- | --- | --- | --- | --- | --- |
| **Parameter** | **Group** | **Pre**  **(mean ± SD)** | **Post**  **(mean ± SD)** | **p-value; Cohen’s *d* [95% CI]** |  | **Mean difference**  **(mean ± SD)** | **p-value, q-value**  **Cohen’s *d* [95% CI]** |
| **At 40% of VO_2max_** |  | | | | | | |
| RPE  (A.U.) | HWI  Control | 11.0 ± 1.70  11.4 ± 0.97 | 9.90 ± 2.02  11.3 ± 0.67 | **p =.03**; *r*_rb_ =0.28 [-0.15, 0.65]  p =.39; *r*_rb_ =-0.06 [-0.50, 0.40] |  | -1.10 ± 0.88  -0.10 ± 1.66 | p =.08, q =.15; *r*_rb_ =0.35 [-0.10, 0.70] |
| Rectal temperature (ºC) | HWI  Control | 37.8 ± 0.23  37.6 ± 0.18 | 37.6 ± 0.21  37.6 ± 0.23 | **p <.01**; *d* =-0.99 [-1.69, -0.32]  p =.05; *d* =-0.03 [-0.45, 0.57] |  | -0.22 ± 0.17  0.00 ± 0.13 | **p<.01, q =.001**; *d* =-1.41[-2.31, -0.52] |
| Heart rate  (BPM) | HWI  Control | 148 ± 8.78  151 ± 8.56 | 139 ± 9.50  146 ± 10.8 | **p <.01**; *d* =-1.02 [-1.66, -0.47]  **p <.01**; *d* =-0.49 [-0.98, -0.21] |  | -9.33 ± 6.22  -4.77 ± 3.60 | **p =.03**, q =.08; *d* =-0.89 [-1.84, 0.26] |
| SpO_2_  (%) | HWI  Control | 77.0 ± 5.60  73.6 ± 6.72 | 79.8 ± 4.49  75.4 ± 6.23 | **p <.01**; *d* =0.55 [0.26, 0.83]  **p <.01**; *d* =0.27 [0.13, 0.51] |  | 2.78 ± 1.89  1.75 ± 1.08 | p =.08, q =.15; *d* =0.67 [-0.45, 1.54] |
| V̇O_2_  (L.min^-1^) | HWI  Control | 1.63 ± 0.28  1.76 ± 0.23 | 1.55 ± 0.30  1.60 ± 0.24 | **p =.02**; *d* =-0.28 [-0.51, -0.05]  **p =.01**; *d* =-0.66 [-1.38, -0.33] |  | -0.08 ± 0.11  -0.16 ± 0.16 | p =.88, q =.85; *d* = 0.55 [-0.37, 1.39] |
| RER  (ratio) | HWI  Control | 1.09 ± 0.08  1.01 ± 0.07 | 1.14 ± 0.21  1.06 ± 0.11 | p =.80; *d* =0.29 [-0.42, 0.89]  p =.92; *d* =-0.51 [-0.12, 1.24] |  | 0.05 ± 0.16  0.05 ± 0.10 | p =.24, q =.32; *r*_rb_ =-0.35 [-0.70, 0.10] |
| V̇_E_  (L.min^-1^) | HWI  Control | 54.4 ± 7.97  50.8 ± 6.39 | 49.6 ± 9.06  48.2 ± 5.78 | **p <.01**; *d* =-0.56 [-0.89, -0.31]  p =.99; *r*_rb_ =0.20 [-0.25, 0.55] |  | -4.78 ± 3.28  -2.55 ± 2.52 | **p =.05,** q =.11; *d* =-0.76 [-1.57, 0.18] |
| Tidal volume  (L) | HWI  Control | 2.26 ± 0.53  2.25 ± 0.51 | 2.12 ± 0.44  2.23 ± 0.49 | **p =.03**; *d* =0.29 [0.08, 0.58]  p =.38; *d* =0.04 [-0.86, 0.96] |  | -0.14 ± 0.21  -0.02 ± 0.21 | p =.09, q =.16; *d* =-0.61 [-1.27, 0.47] |
| **At 60% of VO_2max_** |  | | | | | | |
| RPE  (A.U.) | HWI  Control | 13.6 ± 1.78  14.3 ± 0.95 | 12.0 ± 1.33  13.3 ± 0.82 | **p <.01**; *d* =-1.02 [-1.63, -0.55]  **p <.01**; *r*_rb_ =0.28 [-0.15, 0.65] |  | -1.60 ± 1.43  -1.00 ± 0.82 | p =.17, q =.25; *d* =-0.51 [-1.33, 0.48] |
| Rectal temperature (ºC) | HWI  Control | 37.9 ± 0.23  37.7 ± 0.26 | 37.6 ± 0.22  37.7 ± 0.19 | **p <.01**; *r*_rb_ =0.6 [0.25, 0.85]  p =.34; *d* =-0.08 [-0.68, 0.37] |  | -0.26 ± 0.20  -0.02 ± 0.15 | **p<.01, q =.001**; *r*_rb_ =-0.58 [-0.85, -0.2] |
| Heart rate  (BPM) | HWI  Control | 165 ± 8.49  166 ± 8.96 | 154 ± 8.26  161 ± 8.62 | **p <.01**; *d* =-1.3 [-2.13, -0.62]  **p <.01**; *d* =-0.53 [-1.1, -0.18] |  | -10.9 ± 8.13  -4.67 ± 4.46 | **p =.02**, q =.07; *d* =-0.95 [-1.84, 0.11] |
| SpO_2_  (%) | HWI  Control | 73.6 ± 4.00  72.3 ± 5.92 | 77.1 ± 3.92  73.6 ± 5.21 | **p <.01**; *d* =0.87 [0.62, 1.08]  **p =.01**; *d* =0.22 [0.04, 0.47] |  | 3.46 ± 1.32  1.25 ± 1.43 | **p <.01, q =.001**; *d* =1.6 [0.65, 2.51] |
| V̇O_2_  (L.min^-1^) | HWI  Control | 2.12 ± 0.39  2.23 ± 0.46 | 1.78 ± 0.51  1.91 ± 0.43 | **p =.02**; *d* =-0.50 [-0.97, -0.14]  **p =.02**; *d* =-0.68 [-1.44, -0.23] |  | -0.23 ± 0.29  -0.31 ± 0.25 | p =.74, q =.82; *d* =0.29 [-0.75, 1.18] |
| RER  (ratio) | HWI  Control | 1.13 ± 0.08  1.07 ± 0.07 | 1.23 ± 0.29  1.19 ± 0.20 | p =.87; *d* =0.45 [-0.10, 1.03]  p =.96; *d* =0.76 [0.02, 1.36] |  | 0.10 ± 0.26  0.12 ± 0.18 | p =.43, q =.51; *d* =-0.08 [-1.08, 0.91] |
| V̇_E_  (L.min^-1^) | HWI  Control | 72.1 ± 6.78  69.9 ± 9.49 | 64.0 ± 6.32  66.4 ± 8.57 | **p <.01**; *d* =-1.24 [-2.57, -0.66]  **p <.01**; *d* =-0.38 [-0.79, -0.11] |  | -8.11 ± 5.40  -3.45 ± 2.95 | **p =.02**, q =.07; *d* =-1.07 [-2.0, -0.15] |
| Tidal volume  (L) | HWI  Control | 2.48 ± 0.62  2.51 ± 0.48 | 2.24 ± 0.58  2.49 ± 0.44 | **p =.02**; *d* =0.39 [-0.53, 1.29]  p =.38; *d* =0.03 [-0.89, 0.95] |  | -0.24 ± 0.31  -0.01 ± 0.15 | **p =.03,** q =.08; *d* =-0.89 [-1.76, 0.13] |
